# Spatiotemporal Assessment of PM_2.5_-Related Economic Losses from Health Impacts during 2014–2016 in China

**DOI:** 10.3390/ijerph15061278

**Published:** 2018-06-16

**Authors:** Yang Yang, Liwen Luo, Chao Song, Hao Yin, Jintao Yang

**Affiliations:** 1 School of Geoscience and Technology, Southwest Petroleum University, Chengdu 610500, China; swpu_yangy@126.com (Y.Y.); swpu_ssdata_lab@126.com (L.L.); believeyjt@163.com (J.Y.); 2 State Key Laboratory of Environmental Simulation and Pollution Control, School of Environment, Beijing Normal University, Beijing 100875, China; yinhao@mail.bnu.edu.cn; 3 Department of Geography, Dartmouth College, Hanover, NH 03755, USA; 4 Department of Planning, Danish Centre for Environmental Assessment, Aalborg University, Rendsburggade 14, 9000 Aalborg, Denmark

**Keywords:** PM_2.5_, spatiotemporal assessment, health impact, economic loss, China

## Abstract

*Background*: Particulate air pollution, especially PM_2.5_, is highly correlated with various adverse health impacts and, ultimately, economic losses for society, however, few studies have undertaken a spatiotemporal assessment of PM_2.5_-related economic losses from health impacts covering all of the main cities in China. *Methods*: PM_2.5_ concentration data were retrieved for 190 Chinese cities for the period 2014–2016. We used a log-linear exposure–response model and monetary valuation methods, such as value of a statistical life (VSL), amended human capital (AHC), and cost of illness to evaluate PM_2.5_-related economic losses from health impacts at the city level. In addition, Monte Carlo simulation was used to analyze uncertainty. *Results*: The average economic loss was 0.3% (AHC) to 1% (VSL) of the total gross domestic product (GDP) of 190 Chinese cities from 2014 to 2016. Overall, China experienced a downward trend in total economic losses over the three-year period, but the Beijing–Tianjin–Hebei, Shandong Peninsula, Yangtze River Delta, and Chengdu-Chongqing regions experienced greater annual economic losses. *Conclusions*: Exploration of spatiotemporal variations in PM_2.5_-related economic losses from long-term health impacts could provide new information for policymakers regarding priority areas for PM_2.5_ pollution prevention and control in China.

## 1. Introduction

Rapid industrialization and urbanization have made China one of the fastest growing economies in the world [1], although this growth is having negative effects on both the environment and public health. PM_2.5_ refers to particulate matter suspended in the atmosphere with an aerodynamic diameter ≤ 2.5 μm. It is suspended in the air for a long time, has a small particle size and a large surface area, is very active, carries a lot of poisonous substances, enters the body mainly through the respiratory tract, and penetrates deep into the tracheobronchial and alveolar regions [2]. Short-term and long-term exposure to PM2.5 is closely correlated with a range of acute and chronic health impacts, such as respiratory diseases [3], cardiovascular diseases [4,5], lung cancer [6,7,8], nervous system diseases [9], and congenital heart defects [10]. These negative health effects produce a heavy economic burden, including increased health expenditure, loss of working days, and reduced labor supply [11]. Therefore, it is important for the scientific planning of China’s economic development to conduct a spatiotemporal evaluation of the health-related economic losses caused by PM_2.5_ pollution in China. 

Extensive research regarding PM_2.5_-related economic losses has highlighted the severity of the problem in China today. Health-related economic losses caused by PM_2.5_ pollution totaled USD 0.76–1.04 billion in 2014 and USD 0.68–0.99 billion in 2015 in Beijing [12]. In the Beijing–Tianjin–Hebei region of China, the estimated economic losses caused by PM_2.5_ in 2009 totaled USD 27.1 billion, or 4.7% of regional gross domestic product (GDP) [13]. The estimated economic losses in the Yangtze River Delta region in 2010 totaled USD 3.5 billion, or around 0.2% of regional GDP [14]. It is estimated that in 2013, losses of USD 4.4 billion were caused by high PM_2.5_ pollution at the provincial level, which can be assessed as 54 percent of the total economic loss during 2001–2013 equivalently [15].

Most previous studies have examined cities and regions with high pollution, such as the Beijing–Tianjin–Hebei region [13], Beijing [12,16], The Yangtze River Delta region [14], Shanghai [17], and 74 major cities in China [18]. However, in addition to small-scale studies (big cities and areas with high pollution), it is equally important to study general trends via national-level studies. Only a few studies have analyzed China as a whole when evaluating either PM_2.5_-related health impacts or economic losses [15,19,20], but these have been at the provincial level, rather than the city level.

Previous studies have also examined the spatial distribution of PM_2.5_-related economic losses from health impacts. In Beijing, the external costs in the central and southern areas were higher than those in the northern districts [16]. In the southwest of Taiwan, the cities of New Taipei and Kaohsiung experienced the greatest numbers of deaths attributable to PM_2.5_ [21]. Another study reported that high levels of premature mortality were found in the central and eastern parts of China, with the highest levels located in northern China and the Yangtze River Delta region [22]. However, these studies were limited to a sole space dimension in a single city or region. Moreover, some existing studies have applied time-series analyses to study changing trends in PM_2.5_-related health impacts on a national scale [23,24], yet few studies have examined the influence of the combination of spatial and temporal effects on PM_2.5_-related economic losses from health impacts at the city level.

Thus, this study aims to fill the gap in terms of the spatiotemporal evaluation of nationwide economic losses from health impacts as a result of PM_2.5_ pollution at the city level for the period 2014–2016. Compared to previous studies focusing on provinces or a few large cities, our city-level analyses can give fine-scale spatial differences of health impacts and costs of PM_2.5_ to facilitate policymaking. For example, we can provide detailed information by detecting and mapping highly polluted areas to mitigate air pollution. We used real-time air-quality data from 190 major Chinese cities, as well as socioeconomic and population data, from 2014 to 2016. In addition, we used log-linear (LL) exposure–response functions to evaluate long-term PM_2.5_-related health impacts across China at the city level, using both the value of a statistical life (VSL) and amended human capital (AHC) approaches to determine the upper and lower bounds of mortality-related economic losses from health impacts as a result of PM_2.5_, and cost of illness (COI) to measure morbidity-related economic losses including hospital admission costs, medical costs, and lost working days [25]. In addition, Monte Carlo simulation was used to help determine the uncertainty levels.

The study had three goals: (i) to estimate economic losses from health impacts as a result of PM_2.5_ pollution across China in the period 2014–2016 using two different bounds; (ii) to reveal the spatiotemporal dynamics of PM_2.5_-related economic losses from 2014 to 2016 across China at the city level; and (iii) to quantitatively address the uncertainty of the modeling. The results provide new information for policymakers regarding priority areas in terms of air pollution prevention and control in response to the growing importance of environmental protection in China.

## 2. Materials and Methods 

### 2.1. PM_2.5_ Concentration and Socio-Economic Data

Rapid urbanization and modernization in China over the past few decades have contributed to a significant increase in both urban population and associated carbon emissions [15,19]. The average annual PM_2.5_ concentration was more than 47 µg/m^3^ in 2016, which was more than four times higher than the level specified in the WHO guidelines (10 µg/m^3^) [26,27]. In this study, monthly PM_2.5_ concentration data were retrieved from 1566 monitoring stations in 190 cities operated by China’s online air quality monitoring and analysis platform for the period from January 2014 to December 2016 (http://www.aqistudy.cn/). Our socio-economic data are from the 2014–2016 national economic and social development bulletins of 190 cities, including population, per capita GDP, and per capita disposable income. Since the socio-economic data is collected by year, we obtained monthly PM_2.5_ concentration in each city due to accessibility, processed monthly the PM_2.5_ data into the annual average to match the socio-economic data. 

### 2.2. Health Impact Assessment

A recent study has reported the assessment of PM_2.5_-related health effects applying comprehensive meta-analysis [16]. In the analysis, the authors considered the health impact of various diseases and utilized the 10th version of the International Classification of Diseases report (ICD-10) to avoid double counting of PM_2.5_ health impacts, and they do so because the simple sum of all health costs together without a prior proper classification will lead to overlaps and double counting. In addition, they referred to 24 research results (see Supplementary Materials S1), many of which are the latest research results. Exposure-response coefficients and baseline incidences were reviewed from these literature, giving priority to studies performed in China to improve the exposure-response accuracy. Last, both of our study areas are in China, and within similar research period. Taking the above factors into account, we applied the corresponding methodology and coefficients in this research. 

Health impacts were classified into two categories: mortality and morbidity. Furthermore, they were categorized as either chronic or acute impacts. PM_2.5_ health impacts were estimated for cardiovascular mortality, cardiopulmonary mortality, lung cancer mortality, respiratory mortality, chronic bronchitis, cardiovascular hospitalization, acute bronchitis, and asthma attacks.

The occurrence of either disease or death is a low-probability event, and exhibits a standard Poisson distribution. The risk ratio model in the Poisson regression model forms the basis of current epidemiological studies on air pollution [28]. Therefore, exposure–response functions can be represented by Equations (1) and (2):

(1) $$E_{i}=E_{0i}\times e^{\beta_{i}\left(C-C_{0}\right)}$$

(2) $$HI_{ij}=P_{j}\times \left(E_{i}-E_{0}\right)=P_{j}\times E_{0i}\times \left[e^{\beta_{i}\left(C-C_{0}\right)}-1\right]$$

where *HI_ij_* denotes the health impact *i* in city *j* under pollution level C, *P_j_* is the exposed population in city *j*, *E_i_* refers to the incidence of health end point *i* under pollution level C, *E*_0*i*_ is the baseline incidence of health end point *i* of the affected population, which represents the change in incidence of a health impact per *i* µg/m^3^ increments of PM_2.5_, *β_i_* is the exposure–response coefficient of health end point *i*, *E*_0*i*_ and *β_i_* are derived from Yin’s meta-analysis results (see Supplementary Material S1), *C* refers to the average annual PM_2.5_ concentration level (µg/m^3^), and *C*_0_ is the baseline PM_2.5_ concentration. We use *C*_0_ = 10 µg/m^3^, as recommended by the WHO [26], as the baseline concentration.

### 2.3. Economic Loss Evaluation

In this study, we applied both the VSL and AHC approaches to define the upper and lower bounds of mortality-related economic losses as a result of PM_2.5_, and COI was used to estimate morbidity-related economic losses. The following estimation process follows the research conducted by Yin et al [16].

The VSL refers to the benefits of risk reduction by eliciting individuals’ preferences for small changes in risk and income [29]. We converted VSL for China using Equation (3) [30]:

(3) $$VSL_{j}=VSL_{o}\times {\left(\frac{Income_{j}}{Income_{0}}\right)}^{Elasticity}$$

where *VSL*_0_ is the threshold for the VSL and *Income*_0_ is the threshold for per capita disposable income. We selected USD 248,172, which was obtained from the latest assessment of air pollution in China, as *VSL*_0_ [31], and USD 1939, which was obtained from the National Bureau of Statistics of the People’s Republic of China (NBSC), as *Income*_0_ [32]. *Income*_j_ is the per capita disposable income in city *j*. Because the income elasticity of the VSL is a positive value, we set this to 0.8, as recommended by the Organization for Economic Co-operation and Development [33].

The AHC approach uses per capita GDP to measure the human capital loss as a result of premature death, was used based on Equation (4) [34]:

(4) $$HCL_{j}={\sum_{y=1}^{t}GDP_{j}}_{y}^{dv}=GDP_{j0}\times \sum_{y=1}^{t}\frac{{\left(1+a\right)}^{y}}{{\left(1+r\right)}^{y}}$$

where $GDP_{j}{}_{y}^{dv}$ is the discounted value of per capita GDP in year y in city *j*, *t* is the average number of life-years lost as a result of PM_2.5_ pollution (assumed to be 10 years), *GDP_j_*_0_ is the per capita GDP in base year in city *j*, *a* is the per capita GDP growth rate, and *r* is the social discount rate. We set *a* to 0.07 and *r* to 0.08 in accordance with the reports of the NBSC [35].

COI quantifies the cost of certain diseases in terms of medical treatment costs, hospitalization costs, and productivity loss [25]. COI is used to estimate the morbidity-related economic loss as a result of PM_2.5_ pollution. The costs associated with asthma attacks, acute bronchitis, and cardiovascular-related hospital admissions were obtained from health and family planning career development statistical bulletin for the period during 2014–2016 [36]. We convert the costs related to the treatment of chronic diseases using disability weights. The weight of chronic bronchitis was estimated to be 0.055 of the VSL suggested by Hammitt and Zhou [37].

The total economic loss is estimated using the following equations:

(5) $$EC_{ij}=HI_{ij}\times Cost_{j}$$

(6) $$EC_{total}=EC_{mortality}+EC_{morbidity}$$

where *EC_ij_* is the economic loss from health impact *i* in city *j*, *Cost_j_* is the economic cost per case, *EC_total_* is total economic losses from health impacts, and *EC_mortality_* and *EC_morbidity_* are the economic losses from mortality and morbidity, respectively.

Given the upper and lower bounds of mortality-related economic losses calculated by VSL and AHC respectively, we get the range of estimates according to Equation 6. In upper bounds, the total economic loss equals to the sum of mortality-related economic losses calculated by VSL and morbidity-related economic loss. For lower bound, the total economic loss equals to the sum of mortality-related economic loss calculated by AHC and morbidity-related economic loss.

### 2.4. Uncertainty Analysis

Uncertainty analysis is a measure of model performance, and Monte Carlo simulations are a way of using the probability distributions of input variables to assess the probability distributions of output variables, hence this method is widely used in probabilistic risk assessment. In this study, Crystal Ball software was used to calculate the probability distributions and confidence intervals of PM_2.5_-related economic losses and the Monte Carlo method was used to calculate uncertainty [38]. Uncertainty is defined as an acceptable error of ±5% in relation to each output parameter (i.e., uncertainty of the equation to calculate output value within the range of 5% from the expected predicted value). 

In the process of assessing PM_2.5_-related economic losses from health impacts, the first uncertainty arose in relation to PM_2.5_ exposure levels, which are influenced by factors such as climate and geographic conditions. Moreover, the exposure–response coefficients were derived from the results of a meta-analysis of previous epidemiological studies, and thus we should also consider their uncertainty. In addition, health costs as a result of PM_2.5_ exposure differ among the various studies. In terms of the medical costs of various diseases, these differences stem from the individuals’ physical condition and hospitalization costs. Regarding the VSL, the uncertainty is caused by differences in the willingness to pay for risk prevention, thus the uncertainty of economic losses per case for different health end points was also accounted for in the assessment. Therefore, the uncertainty analysis covered PM_2.5_ exposure concentration, exposure–response coefficients, and health-related economic loss per case. In addition, AHC values were calculated based on the per capita GDP, and because the uncertainty of this estimate was unknown, the uncertainty of economic losses from mortality estimated using the AHC approach was not included in this study. 

## 3. Results

### 3.1. Spatial Distribution of PM_2.5_

**Figure 1:**
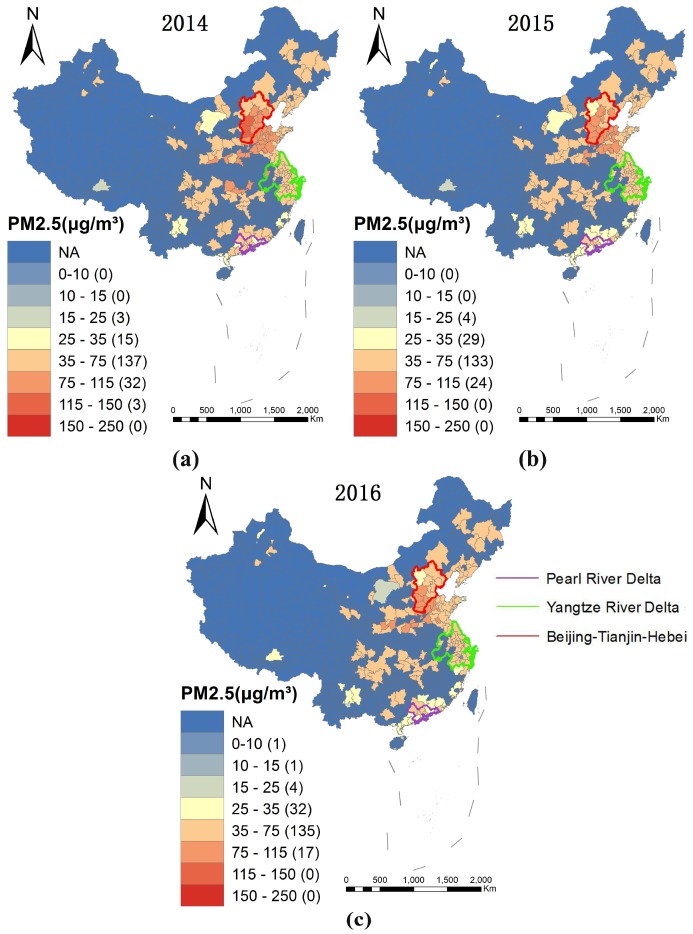
Spatial distribution of PM_2.5_ in 2014 (**a**); 2015 (**b**); and 2016 (**c**).

Figure 1 shows the spatial distribution of PM_2.5_ in 2014 (a), 2015 (b), and 2016 (c) across China. From the perspective of economic development, spatial analysis reveals that the Beijing–Tianjin–Hebei region experienced the highest levels of PM_2.5_ pollution, followed by the Yangtze River Delta region and the Pearl River Delta region, which is consistent with the results reported in the “2016 China Environmental Status Bulletin” [27]. 

PM_2.5_ pollution areas displayed similarities across the period, high-polluted areas were concentrated in the central and eastern districts, especially in the Beijing–Tianjin–Hebei, Central Henan, Shanxi, and Western Shandong Peninsula, and less-polluted areas includes Lhasa, Chifeng, Yunnan, Pearl River Delta, and the Western Taiwan Straits. Figure 1 also indicates that the number of high pollution (>75 µg/m^3^) areas has declined, and “2014–2016 China Environmental Status Bulletin” has reported that the annual PM_2.5_ concentration across China has decreased from 62 μg/m^3^ in 2014, and 50 μg/m^3^ in 2015, to 47 μg/m^3^ in 2016 [27]. Even though the overall PM_2.5_ pollution was being controlled, the level of pollution remained serious between 2014 and 2016, all cities exceeded WHO guidelines and approximately 71 % of the cities are 35–75 μg/m^3^. 

### 3.2. PM_2.5_ Health Impact

Table 1 shows a downward trend in the overall affected population, reducing by 13.8%. The average annual decline in PM_2.5_-related premature deaths was 11.8%, and the incidence of asthma attacks was higher than that of other diseases.

**Figure 2:**
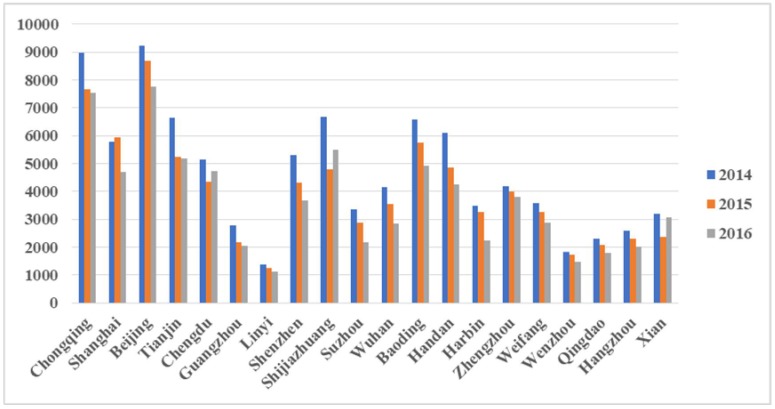
PM_2.5_-related health impacts (all-cause mortality) in 20 major cities in China during the period 2014–2016.

Figure 2 displays the PM_2.5_-related health impacts (all-cause mortality) in 20 major Chinese cities during the period 2014–2016. Beijing was ranked first, followed by Chongqing, Shijiazhuang, Tianjin, and Baoding. Table S3 provides more detailed information in relation to all 190 cities studied.

**Figure 3:**
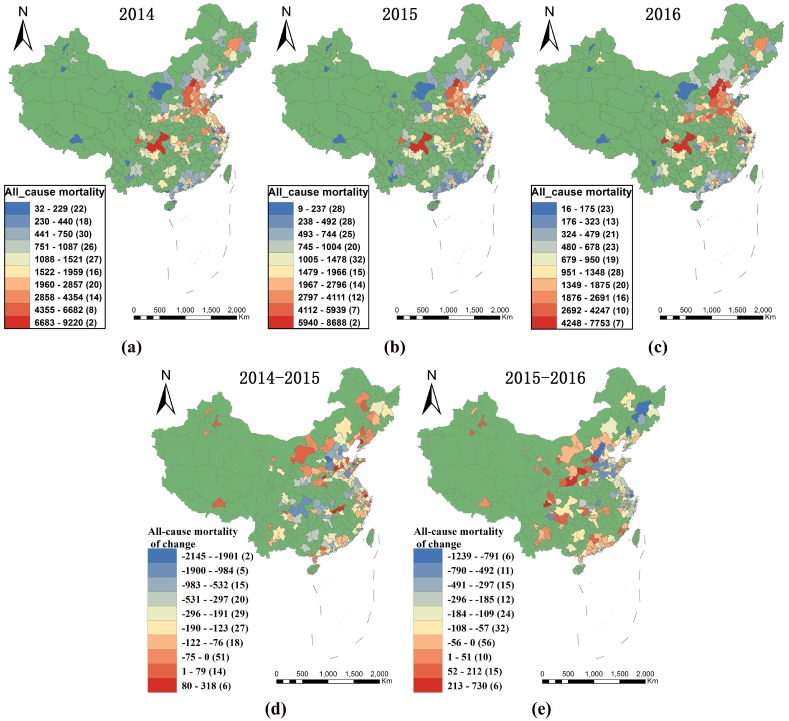
Spatial distribution of PM_2.5_-related health impacts (all-cause mortality) in 2014 (**a**); 2015 (**b**); and 2016 (**c**). Distribution of changes in PM_2.5_-related health impacts (all-cause mortality) for the periods 2014–2015 (**d**) and 2015–2016 (**e**).

Figure 3 displays the distribution of PM_2.5_-related health impacts (all-cause mortality) during the period 2014–2016 and changes in the spatial distribution over the period. PM_2.5_-related premature deaths in each city characterize the health impact for that area in that year. The highest-impacted areas displayed similarities across the period and were concentrated in the central and eastern districts, especially in the Beijing–Tianjin–Hebei, Central Henan, Shanxi, Western Shandong Peninsula, Yangtze River Delta, and Chengdu–Chongqing regions, while the less-impacted areas were mainly concentrated in the northwest, including Lhasa, Inner Mongolia, Yunnan, Guizhou, and the Western Taiwan Straits, as well as in southern China. However, compared with 2014–2015 values, the areas showing the greatest improvement in 2015–2016 were concentrated in the Beijing–Tianjin–Hebei region, excluding the Shijiazhuang, Shandong Peninsula, Yangtze River Delta, and northeast regions, while the worst-impacted areas shifted from Western Inner Mongolia and the northeast, Central Henan, Western Shandong Peninsula, and Yangtze River Delta regions, to the central Shaanxi Plain, the southeast, Shanxi regions, the middle reaches of the Yangtze River, and the Western Taiwan Straits.

### 3.3. Economic Loss of Health Impacts

Table 2 shows that overall, China experienced a slight downward trend in economic losses between 2014–2015 and 2015–2016. The average economic loss was between 0.3% (AHC) and 1% (VSL) of the total GDP of 190 Chinese cities from 2014 to 2016. The economic losses estimated using the AHC method were around 26% of those estimated using the VSL method. Economic losses as a result of all-cause mortality accounted for around 80% (AHC) to 95% (VSL) of total economic losses. Table S4 shows more detailed information for the 190 cities studied. 

**Figure 4:**
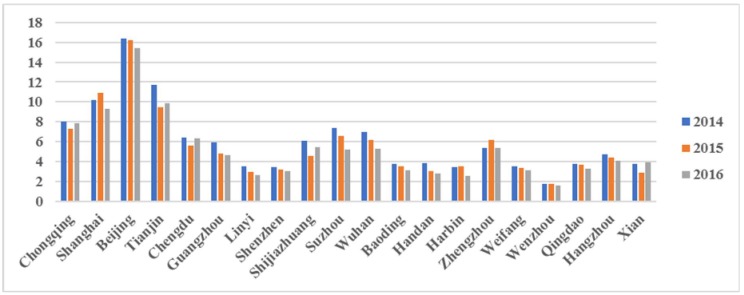
PM_2.5_-related economic losses as a result of health impacts in 20 Chinese major cities in the lower bounds during the period 2014–2016.

**Figure 5:**
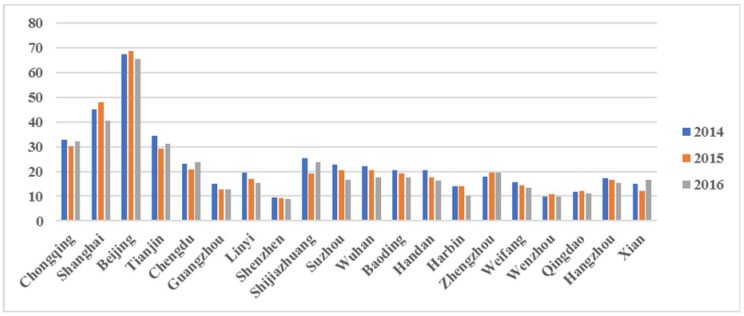
PM_2.5_-related economic losses as a result of health impacts in 20 Chinese major cities in the upper bounds during the period 2014–2016.

Figure 4 and Figure 5 shows that, similar to the PM_2.5_-related health impacts, Beijing was ranked first in terms of health-related economic losses, followed by Tianjin, Shanghai, and Chongqing. From a regional perspective, of the 20 major cities that experienced the greatest economic losses, seven were in southern China and six were in northern China. However, the rankings of Guangzhou, Linyi, Shenzhen, and Wenzhou were different in two different bounds. 

**Figure 6:**
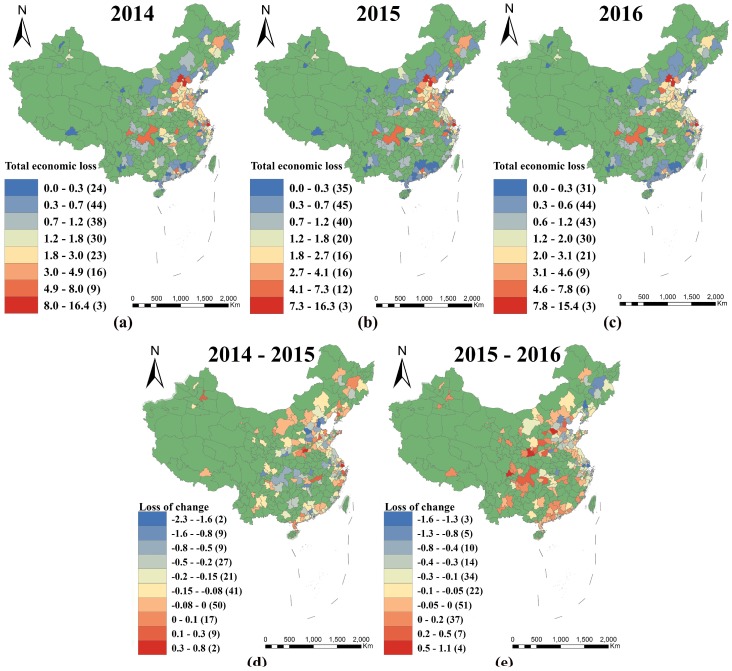
Spatial distribution of PM_2.5_-related economic losses in the lower bounds in 2014 (**a**); 2015 (**b**); and 2016 (**c**). Distribution of changes in PM_2.5_-related economic losses in the lower bounds for the periods 2014–2015 (**d**) and 2015–2016 (**e**).

**Figure 7:**
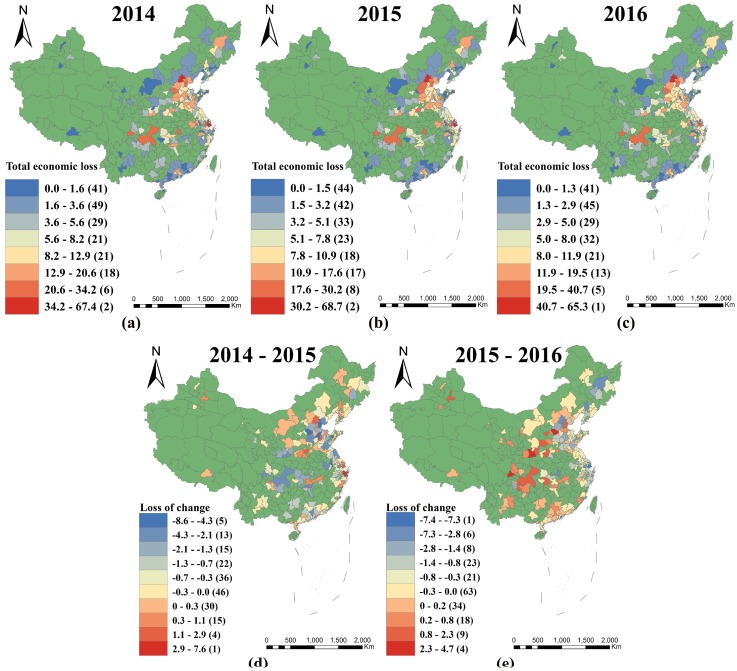
Spatial distribution of PM_2.5_-related economic losses in the upper bounds in 2014 (**a**); 2015 (**b**); and 2016 (**c**). Distribution of changes in PM_2.5_-related economic losses in the upper bounds for the periods 2014–2015 (**d**) and 2015–2016 (**e**).

Figure 6 and Figure 7 show the distribution of PM_2.5_-related economic losses during the period 2014–2016 and changes in the spatial distribution over the years, respectively. The highest-ranked areas displayed similarities across the period and were concentrated in the central and eastern districts, especially in the South Beijing–Tianjin–Hebei, Central Henan, Western Shandong Peninsula, Yangtze River Delta, and Chengdu–Chongqing regions, the middle reaches of the Yangtze River, and the Guangzhou region, while the lower-ranked areas were mainly concentrated in the northwest, Inner Mongolia, Lhasa, North Beijing–Tianjin–Hebei, Yunnan, Guizhou, southern China (excluding Guangzhou), central Shaanxi Plain, Shanxi, Eastern Shandong Peninsula, northeast, and Western Taiwan Straits regions.

Furthermore, China experienced a downward trend in economic losses during the period from 2014 to 2016. However, compared with 2014–2015 values, the areas showing the most improvement in 2015–2016 were concentrated in the Beijing, Baoding, Chifeng, Shandong Peninsula, Yangtze River Delta, and northeast regions, while the worst-impacted regions shifted from Western Inner Mongolia, Central Henan, and the Yangtze River Delta, to Shijiazhuang, Tianjin, Henan, Shanxi, the central Shaanxi Plain, Chengdu–Chongqing, Yunnan, Guizhou, the middle reaches of the Yangtze River, the Western Taiwan Straits, and southern China.

### 3.4. Uncertainty Analysis

**Figure 8:**
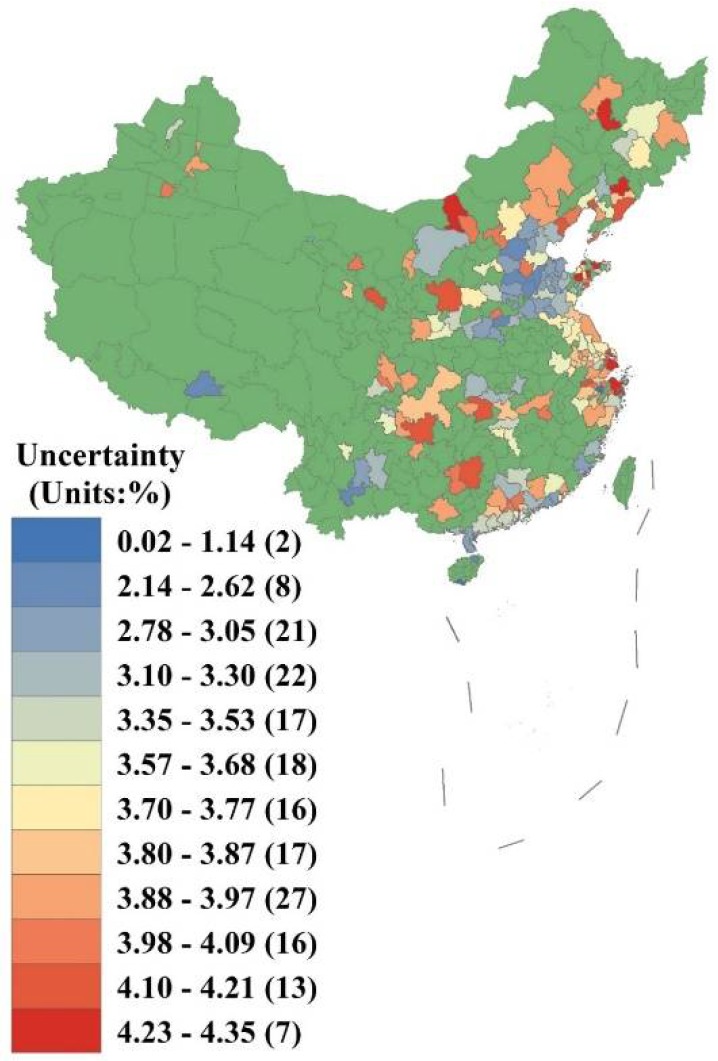
Spatial distribution of uncertainty in relation to health-related economic losses using the value of a statistical life (VSL).

Figure 8 shows the spatial distribution of uncertainty in relation to the health-related economic losses calculated using the VSL method for 190 cities. It can be seen that overall uncertainty ranges from 0.02% to 4.35%. The northwest, Chengdu–Chongqing, middle reaches of the Yangtze River, Guizhou, Guangxi, Yangtze River Delta, Eastern Shandong Peninsula, Central Inner Mongolia, and northeast regions have the highest levels of uncertainty, indicating that the results in these areas are somewhat imprecise. In contrast, the Beijing–Tianjin–Hebei, Central Henan, Western Shandong Peninsula, Lhasa, Yunnan, Pearl River Delta (excluding Guangzhou), and Western Taiwan Straits regions have lower levels of uncertainty, indicating that the results are more reliable. 

## 4. Discussion

China experienced a downward trend in economic losses as a result of health impacts during the period 2014–2016, but average annual economic losses remained high, especially in the central and southern areas of China, such as the Beijing–Tianjin–Hebei, Shandong, Yangtze River Delta, and Chengdu–Chongqing regions. However, compared with 2014–2015, the areas showing the most improvement in 2015–2016 are concentrated in the Beijing, Baoding, Chifeng, Shandong Peninsula, Yangtze River Delta, and northeast regions, while the worst-impacted regions shifted from Western Inner Mongolia, Central Henan, and the Yangtze River Delta to Shijiazhuang, Tianjin, Henan, Shanxi, the central Shaanxi Plain, Chengdu–Chongqing, Yunnan, Guizhou, the middle reaches of the Yangtze River, the Western Taiwan Straits, and southern China. The reasons for this spatial distribution include pollution emissions, meteorological conditions, and population density. Meanwhile, China has experienced demographic dividend, and is now suffering low fertility and an ageing population, which is leading to a scarcity of labor, and increasing PM_2.5_ pollution could make this situation even worse. The temporal trend indicates that overall, air pollution in China has gradually improved in recent years, thanks to a series of aggressive measures adopted by the Chinese government and the relevant urban management authorities, the implementation of measures introduced in 2016 aimed at the prevention and control of air pollution in Beijing during the period 2016–2017, and the Environmental Protection Tax Law of the People’s Republic of China introduced on 1 January 2018, whereby the tax deduction related to reductions in environmental pollution shall be USD 0.19 to USD 1.87 per pollutant equivalent. Moreover, the changes in the spatial distribution show that air pollution control measures were more effective in key cities and regions such as Beijing, Baoding, Chifeng, the Shandong Peninsula, the Yangtze River Delta, and the northeast. In contrast, regions such as Shijiazhuang, Tianjin, Henan, Shanxi, the central Shaanxi Plain, Chengdu–Chongqing, Yunnan, Guizhou, the middle reaches of the Yangtze River, the Western Taiwan Straits, and southern China were clearly more committed to pursuing economic development while ignoring environmental governance issues. 

The results of this study show that 21.7 million people suffered a health impact as a result of PM_2.5_ pollution and there were more than 0.28 million premature deaths (about 0.03% of the total population) in China in 2014, 18.1 million people suffered a health impact and there were 0.24 million premature deaths (again, about 0.03% of the total population) in 2015, and 16.1 million people suffered a health impact and there were 0.22 million premature deaths (about 0.02% of the total population) in 2016. Acute bronchitis and asthma attacks were the health end points affecting most people. Our results are in general agreement with those of previous studies, e.g., premature deaths in the Pearl River Delta accounted for approximately 0.02% of the total population in 2012 [39], and an assessment of China’s PM_2.5_ health impacts on people of all ages indicated that premature deaths accounted for 0.09% of the total population in 2013 [40]. In addition, the results varied from country to country. In India, the average annual health burden was estimated to be 5700 premature deaths (0.16% of the total population) during the period 2001–2015 [41], while another study showed that 3300 premature deaths in Nagpur in 2013 could be attributed to ambient PM_2.5_ pollution [42]. One study found that PM_2.5_ pollution caused 37,000 deaths in 27 Southeast and East Asian countries in 2009 [43]. In the United States (US), premature deaths as a result of PM_2.5_ pollution were estimated to account for 0.04% of the total population in 2005 [44]. In Sweden, a recent study estimated that there were 3500 premature deaths per year as a result of PM_2.5_ pollution [45]. These results illustrate that the mortality rates in China and India were much higher than those in Europe and the US. We also compared the findings with those of the integrated exposure–response model in China, GBD 2015 which indicated that PM_2.5_ pollution caused 1,108,000 deaths [46]. Total premature mortality in 190 cities in China as a result of PM_2.5_ pollution was estimated to be 722,370 in the period 2014–2015 [47]. Another study showed that approximately 1,126,000 deaths were caused by PM_2.5_ pollution in 2015, and that this figure fell to 1,092,000 in 2016 [24]. Our figures are lower than those presented in these studies, which indicates that the LL model may underestimate the health impacts of PM_2.5_ pollution. In summary, PM_2.5_ concentrations, baseline levels, exposure–response coefficients, exposure–response functions, and choice of health endpoints all influence the results found by the various studies.

The results also indicate that total economic losses as a result of PM_2.5_-related health impacts ranged from USD 31.79 billion (AHC) to USD 121.92 billion (VSL) or around 0.36% to 1.36% of regional GDP in China in 2014, from USD 28.7 billion (AHC) to USD 111.78 billion (VSL) or around 0.3% to 1.18% of regional GDP in 2015, and from USD 26.74 billion (AHC) to USD 107.2 billion (VSL) or around 0.26% to 1.06% of regional GDP in 2016. Similar conclusions can be found in the existing studies. One study focusing on Beijing reported that lost regional GDP as a result of PM_2.5_ pollution was between 0.3% (AHC) and 0.9% (VSL) in 2012 [16]. Another study found that economic losses from health impacts as a result of PM_2.5_ pollution accounted for between 0.11% (AHC) and 0.4% (VSL) of regional GDP in 2014 [48], while another study predicted future health-related economic losses and indicated that China’s health expenditure will reach US$ 25.2 billion (approximately 2% of GDP) if pollution is not controlled in 2030 [19]. Furthermore, economic losses as a result of premature deaths accounted for around 80% (AHC) to 95% (VSL) of total economic losses. Our results are in general agreement with those of previous studies, which estimated economic losses of 88.4% in 111 Chinese cities in 2004 [49], 90% in China in 2010 [50], and 80% in Beijing in 2012 [16]. In the above comparisons, we actually found that our health economic losses are slightly higher, especially those estimates calculated by VSL. Most of the above studies estimate mortality costs or morbidity related costs without focusing on the health impacts classification, and used different environmental valuation methods, which could explain this difference. Therefore, when these variations are taken into consideration, the results of this study are basically reasonable. 

This study helps to fill the gap in the literature on spatiotemporal evaluation of nationwide economic losses from health impacts as a result of PM_2.5_ pollution at the city level for the period 2014–2016. Our results provide new information for policymakers regarding priority areas in relation to air pollution prevention and control given the trend toward increasing environmental protection in China. However, there are several areas of uncertainty. First, our evaluation was based on city-level PM_2.5_ concentrations calculated by averaging the data from all sites, which is the common method used to report daily air quality to the public. However, the uneven distribution of monitoring stations, with more in urban areas and less in suburban and rural areas, renders a simple averaging method less accurate than more complex methods [51], the spatial correlation effects between cities and peripheral areas also need to be further considered. Second, we assume the exposure population is a permanent residential population in this study, while the actual exposure of the population is dynamic. Previous studies compared the differences between dynamic and static population exposure, which illustrated that the static population exposure could underestimate the exposure level by around 20% [52]. Third, the exposure–response coefficients of PM_2.5_, which rely on the results of epidemiology and toxicology research, require further investigation, and there are many other factors involved in the process of building the exposure–response relationship that remain unclear. Moreover, the age distribution of the population was not considered in the evaluation process. Given that previous studies have found that estimated total losses based on the VSL without considering the age distribution of the population were between 2.3 and 2.8 times greater than those obtained after taking the age structure of the population into account [15], our results might overstate the health-related economic losses. Further, the monetary evaluation method also has its limitations. Previous studies did not focus on different regional characteristics such as living habits, the economic environment, and environmental sanitation, and some health end points lack information related to medical costs, lost working time, and related contingent valuation study. Future work should focus on the promotion in these fields continuously. 

Although these limitations exist, this study makes a significant contribution by exploring the spatiotemporal variations in terms of economic losses from health impacts as a result of air pollution in China. The findings of this study have important implications for policymakers regarding priority areas to be addressed in relation to the prevention and control of air pollution given the trend toward increased environmental protection in China. This study reveals the spatiotemporal dynamics of PM_2.5_-related economic losses from health impacts during the period 2014–2016 across China at the city level. Although there is a degree of uncertainty regarding the value of these losses, this uncertainty also applies to annual assessments, hence the overall trend is not affected. Last, the long-term PM_2.5_ health impacts refer to years to decades [53]. Moreover, atmospheric pollutants have a shorter life span, and their diffusion and removal rate are fast. Therefore, our three-year study can reflect the variations of pollution and its impacts.

## 5. Conclusions

We conducted a spatiotemporal assessment of economic losses from health impacts attributed to PM_2.5_ pollution during the period 2014–2016 in 190 Chinese cities. The results provide new information for policymakers regarding priority areas to be addressed in relation to the prevention and control of air pollution in China. The results show that China experienced a decline in total economic losses from health impacts during the period 2014–2016, but average annual losses remained high, and were mainly concentrated in eastern and central districts. The three cities with the highest level of losses during the period 2014–2016 were Beijing, Chongqing, and Tianjin, which reflects the spatial distribution of PM_2.5_ pollution. Thus, these cities should be the primary focus of efforts to implement PM_2.5_ prevention and control measures. In addition, we found that China’s PM_2.5_-related economic losses of health impacts were enormous, for the reason that the average economic loss was 0.3% (AHC) to 1% (VSL) of the total GDP of 190 Chinese cities from 2014 to 2016. It suggests that PM_2.5_ reduction will have significant benefits at both the regional and national scales. 

The study has some limitations. For instance, the differences between urban and rural PM_2.5_ concentrations were unclear, exposed population selected resident population without considering age structure, exposure—response coefficients were based on the results of previous meta-analyses, and not all PM_2.5_-related health impacts were included in the evaluation. However, despite these limitations, this study makes a significant contribution by exploring China’s spatiotemporal variations in terms of economic losses from health impacts as a result of air pollution. The findings of this study have important implications for policymakers regarding the priority areas in terms of the prevention and control of air pollution given the trend toward increasing environmental protection in China.

## Figures and Tables

**Table 1:** Health impacts attributed to PM_2.5_ pollution in China during the period 2014–2016.

Category	2014	2015	2016
All-cause mortality	278,444	238,622	216,164
Cardiovascular mortality	71,058	60,991	55,321
Respiratory mortality	42,590	36,431	32,959
Lung cancer mortality	92,512	78,444	70,557
Cardiovascular hospitalization	1,001,233	851,497	767,387
Chronic bronchitis	185,798	159,366	144,459
Acute bronchitis	1,034,080	881,692	795,978
Asthma attack	19,197,994	15,935,983	14,130,036
Affected population	21,697,549	18,067,160	16,054,024

**Table 2:** Total economic losses from health impacts as a result of PM_2.5_ pollution during the period 2014–2016.

Category	The Health Economic Loss (100 Million Dollar)					
	2014 AHC	2014 VSL	2015 AHC	2015 VSL	2016 AHC	2016 VSL
All-cause mortality	256.32	1157.59	230.89	1061.69	214.16	1018.62
Cardiovascular mortality	65.46	295.48	59.03	271.44	54.84	260.75
Respiratory mortality	39.19	177	35.23	162.02	32.63	155.24
Lung cancer mortality	84.84	383.9	75.7	348.39	69.68	331.86
Cardiovascular hospitalization	12.2	12.2	10.95	10.95	10.28	10.28
Chronic bronchitis	42.48	42.48	39	39	37.44	37.44
Acute bronchitis	0.35	0.35	0.31	0.31	0.29	0.29
Asthma attack	6.57	6.57	5.8	5.8	5.4	5.4
Total economic loss (TEL)	317.93	1219.19	286.97	1117.76	267.38	1072.02
TEL/GDP	0.36%	1.36%	0.30%	1.18%	0.26%	1.06%

## Supplementary Materials

The following tables are available online at http://www.mdpi.com/1660-4601/15/6/1278/s1. Table S1: Source of the health impacts assessment model parameters and the disease data; Table S2: Assumption cells and forecast cells used for uncertainty analysis; Table S3: PM_2.5_-related health impacts and changes in all-cause mortality from 2014 to 2016 in 190 Chinese cities; and Table S4: PM_2.5_-related economic losses from health impacts and changes in total economic losses from 2014 to 2016 in 190 Chinese cities.

## References

[1] International Monetary Fund (IMF) World Economic Outlook Databases. http://www.imf.org/external/ns/cs.aspx?id=28.

[2] Cao C., Jiang W., Wang B., Fang J., Lang J., Tian G., Jiang J., Zhu T.F. (2014). Inhalable microorganisms in Beijing’s PM_2.5_ and PM_10_ pollutants during a severe smog event. Environ. Sci. Technol..

[3] Mo Z., Fu Q., Zhang L., Lyu D., Mao G. (2018). Acute effects of air pollution on respiratory disease mortalities and outpatients in Southeastern China. Sci. Rep..

[4] Dabass A., Talbott E.O., Venkat A., Rager J., Marsh G.M. (2016). Association of exposure to particulate matter (PM_2.5_) air pollution and biomarkers of cardiovascular disease risk in adult NHANES participants (2001–2008). Int. J. Hyg. Environ. Health.

[5] Huang Z., Zhou Y., Lu Y., Duan Y., Tang X., Deng Q., Yuan H. (2017). A Case-Crossover Study between Fine Particulate Matter Elemental Composition and Emergency Admission with Cardiovascular Disease. Acta Cardiol. Sin..

[6] Huang F., Bing P., Wu J., Chen E., Chen L. (2017). Relationship between exposure to PM_2.5_ and lung cancer incidence and mortality: A meta-analysis. Oncotarget.

[7] Shu Y., Zhu L., Yuan F., Kong X., Huang T., Cai Y.D. (2016). Analysis of the relationship between PM_2.5_ and lung cancer based on protein-protein interactions. Comb. Chem. High Throughput Screen..

[8] Badyda A.J., Grellier J., Dąbrowiecki P. (2016). Ambient PM_2.5_ exposure and mortality due to lung cancer and cardiopulmonary diseases in polish cities. Adv. Exp. Med. Biol..

[9] Van Winkle L.S., Bein K., Anderson D., Pinkerton K.E., Tablin F., Wilson D., Wexler A.S. (2015). Biological dose response to PM_2.5_: Effect of particle extraction method on platelet and lung responses. Toxicol. Sci..

[10] Zhang B., Liang S., Zhao J., Qian Z., Bassig B.A., Yang R., Zhang Y., Hu K., Xu S., Zheng T. (2016). Maternal exposure to air pollutant PM2.5 and PM10 during pregnancy and risk of congenital heart defects. J. Expo. Sci. Environ. Epidemiol..

[11] Kjellstrom T., Holmer I., Lemke B. (2009). Workplace heat stress, health and productivity—An increasing challenge for low and middle-income countries during climate change. Glob. Health Action.

[12] Li L., Lei Y., Wu S., Chen J., Yan D. (2017). The health economic loss of fine particulate matter (PM_2.5_) in Beijing. J. Clean. Prod..

[13] Huang D., Zhang S. (2013). Health benefit evaluation for PM_2.5_ pollution control in Beijing-Tianjin-Hebei region of China. China Environ. Sci..

[14] Wang J., Wang S., Voorhees A.S., Zhao B., Jang C., Jiang J., Fu J.S., Ding D., Zhu Y., Hao J. (2015). Assessment of short-term PM_2.5_-related mortality due to different emission sources in the Yangtze River Delta, China. Atmos. Environ..

[15] Mu Q., Zhang S. (2015). Assessment of the Trend of Heavy PM_2.5_ Pollution Days and Economic Loss of Health Effect during 2001–2013. Acta Sci. Nat. Univ. Pekin..

[16] Yin H., Pizzol M., Xu L. (2017). External costs of PM_2.5_ pollution in Beijing, China: Uncertainty analysis of multiple health impacts and costs. Environ. Pollut..

[17] Wu R., Dai H., Geng Y., Xie Y., Masui T., Liu Z., Qian Y. (2017). Economic Impacts from PM_2.5_ Pollution-Related Health Effects: A Case Study in Shanghai. Environ. Sci. Technol..

[18] Lv L., Li H., Yang J. A health-based economic assessment of PM_2.5_ pollution in Chinese major cities. Proceedings of the 2015 International Forum on Energy, Environment Science and Materials.

[19] Xie Y., Dai H., Dong H., Hanaoka T., Masui T. (2016). Economic Impacts from PM_2.5_ Pollution-Related Health Effects in China: A Provincial-Level Analysis. Environ. Sci. Technol..

[20] Wu J., Zhu J., Li W., Xu D., Liu J. (2017). Estimation of the PM_2.5_ health effects in China during 2000–2011. Environ. Sci. Pollut. Res..

[21] Lo W., Shie R., Chan C., Lin H. (2017). Burden of disease attributable to ambient fine particulate matter exposure in Taiwan. J. Formos. Med. Assoc..

[22] Liu J., Han Y., Tang X., Zhu J., Zhu T. (2016). Estimating adult mortality attributable to PM_2.5_ exposure in China with assimilated PM_2.5_ concentrations based on a ground monitoring network. Sci. Total Environ..

[23] Liu M., Huang Y., Ma Z., Jin Z., Liu X. (2017). Spatial and temporal trends in the mortality burden of air pollution in China: 2004–2012. Environ. Int..

[24] Feng L., Ye B., Feng H., Ren F., Huang S. (2017). Spatiotemporal Changes in Fine Particulate Matter Pollution and the Associated Mortality Burden in China between 2015 and 2016. Int. J. Environ. Res. Public Health.

[25] Huang D., Xu J., Zhang S. (2012). Valuing the health risks of particulate air pollution in the Pearl River Delta, China. Environ. Sci. Policy.

[26] WHO WHO Air Quality Guidelines for Particulate Matter, Ozone, Nitrogen Dioxide and Sulfur Dioxide. http://apps.who.int/iris/bitstream/10665/69477/1/WHO_SDE_PHE_OEH_06.02_eng.pdf.

[27] Ministry of Environmental Protection of the People’s Republic of China. http://www.zhb.gov.cn/hjzl/zghjzkgb/lnzghjzkgb/.

[28] Hong C.J., Kan H.D., Chen B.H. (2005). Quantitative evaluation of the atmosphere pollution about urban resident health hazard. J. Environ. Health.

[29] Hammitt J.K. (2000). Valuing Mortality Risk: Theory and Practice. Environ. Sci. Technol..

[30] Hammitt J.K., Robinson L.A. (2011). The income elasticity of the value per statistical life: Transferring estimates between high and low income populations. J. Benefit-Cost Anal..

[31] Xie X. (2010). The Value of Health: Environmental Benefit Assessment Method and Urban Air Pollution Control Strategy. Master’s Thesis.

[32] China 2012 Statistical Yearbook. http://www.stats.gov.cn/tjsj/ndsj/2012/indexeh.htm.

[33] OECD (2012). Mortality Risk Valuation in Environment, Health and Transport Policies.

[34] Hou Q., An X., Wang Y., Tao Y., Sun Z. (2012). An assessment of China’s PM_10_-related health economic losses in 2009. Sci. Total Environ..

[35] China 2016 Statistical Yearbook. http://www.stats.gov.cn/tjsj/ndsj/2016/indexeh.htm.

[36] China Health and Family Planning Commission Statistical Yearbook. http://www.tjcn.org/e/search/result/?searchid=3173.

[37] Hammitt J.K., Zhou Y. (2006). The economic value of air-pollution-related health risks in China: A contingent valuation study. Environ. Resour. Econ..

[38] Abhishek B., Ashok K. (2010). Application of the crystal ball software for uncertainty and sensitivity analyses for predicted concentration and risk levels. Environ. Prog. Sustain..

[39] Jiang X., Hong C., Zheng Y., Zheng B., Guan D., Zhang Q., Gouldson A., He K. (2015). To what extent can China’s near-term air pollution control policy protect air quality and human health? A case study of the Pearl River Delta region. Environ. Res. Lett..

[40] Apte J.S., Marshall J.D., Cohen A.J., Brauer M. (2015). Addressing Global Mortality from Ambient PM_2.5_. Environ. Sci. Technol..

[41] Jain V., Dey S., Chowdhury S. (2017). Ambient PM_2.5_ exposure and premature mortality burden in the holy city Varanasi, India. Environ. Pollut..

[42] Etchie T.O., Sivanesan S., Adewuyi G.O., Krishnamurthi K., Rao P.S., Etchie A.T., Pillarisetti A., Arora N.K., Smith K.R. (2017). The health burden and economic costs averted by ambient PM_2.5_, pollution reductions in Nagpur, India. Environ. Int..

[43] Yorifuji T., Bae S., Kashima S., Tsuda T., Doi H., Honda Y., Kim H., Hong Y.C. (2015). Health Impact Assessment of PM_10_ and PM_2.5_ in 27 Southeast and East Asian Cities. J. Occup. Environ. Med..

[44] Fann N., Lamson A.D., Anenberg S.C., Wesson K., Risley D. (2012). Estimating the National Public Health Burden Associated with Exposure to Ambient PM_2.5_ and Ozone. Risk Anal..

[45] Åström S., Gustafsson M., Tekie H., Sjöberg K. (2015). Quantification of population exposure to NO_2_, PM_2.5_ and PM_10_ and estimated health impacts in sweden 2010. Hum. Exp. Toxicol..

[46] Cohen A.J., Brauer M., Burnett R., Anderson H.R., Frostad J. (2017). Estimates and 25-year trends of the global burden of disease attributable to ambient air pollution: An analysis of data from the Global Burden of Diseases Study 2015. Lancet.

[47] Maji K.J., Arora M., Dikshit A.K. (2017). Burden of disease attributed to ambient PM_2.5_ and PM10 exposure in 190 cities in China. Environ. Sci. Pollut. Res..

[48] Chen L., Shi M., Gao S., Li S., Mao J. (2017). Assessment of population exposure to PM_2.5_ for mortality in China and its public health benefit based on BenMAP. Environ. Pollut..

[49] Zhang M., Song Y., Cai X., Zhou J. (2008). Economic assessment of the health effects related to particulate matter pollution in 111 Chinese cities by using economic burden of disease analysis. Environ. Manag..

[50] OECD Reviewing the Evidence on and Calculating the Cost of the Health Impacts of Air Pollution, in the Cost of Air Pollution: Health Impacts of Road Transport. http://www.oecd-ilibrary.org/environment/the-cost-of-air-pollution_9789264210448–enaccessed31.01.17.

[51] Guo H., Cheng T., Gu X., Wang Y., Chen H. (2017). Assessment of PM_2.5_ concentrations and exposure throughout China using ground observations. Sci. Total Environ..

[52] Beckx C., Int Panis L., Arentze T., Janssens D., Torfs R., Broekx S., Wets G. (2009). A dynamic activity-based population modelling approach to evaluate exposure to air pollution: Methods and application to a Dutch urban area. Environ. Impact Assess. Rev..

[53] Beverland I.J., Cohen G.R., Heal M.R., Carder M., Yap C., Robertson C., Hart C.L., Agius R.M. (2012). A Comparison of Short-term and Long-term Air Pollution Exposure Associations with Mortality in Two Cohorts in Scotland. Environ. Health Perspect..

