# Supplementary material for: Spatiotemporal Assessment of PM2.5-Related Economic Losses from Health Impacts during 2014–2016 in China
Source: Int J Environ Res Public Health. 2018 Jun 16;15(6):1278. doi: 10.3390/ijerph15061278 (PMC6024949; doi:10.3390/ijerph15061278)
Supplement: Supplementary file 1 [file ijerph-15-01278-s001.zip › ijerph-303841-SI.pdf]

SUPPLEMENTARY MATERIALS

Spatiotemporal Assessment of $PM_{2.5}$-Related Economic Losses from Health Impacts during 2014–2016 in China

Yang Yang [1,†], Liwen Luo [1,†], Chao Song [1,2,3,*], Hao Yin [2,4] and Jintao Yang [1]

[1] School of Geoscience and Technology, Southwest Petroleum University, Chengdu 610500, China; swpu_yangy@126.com (Y.Y.); swpu_ssdata_lab@126.com (L.L.); believeyjt@163.com (J.Y.)
[2] State Key Laboratory of Environmental Simulation and Pollution Control, School of Environment, Beijing Normal University, Beijing 100875, China; yinhao@mail.bnu.edu.cn
[3] Department of Geography, Dartmouth College, Hanover, New Hampshire 03755, USA
[4] Department of Planning, Danish Centre for Environmental Assessment, Aalborg University, 14, 9000 Aalborg, Denmark
* Correspondence: songc345@163.com; Tel.: +86-28-8303-7107
† Yang Yang and Liwen Luo contributed equally to this paper

Contents

1. **Source of the health impacts assessment model parameters and the disease data**
2. **Assumption cells and forecast cells used for uncertainty analysis**
3. **$PM_{2.5}$-related health impacts and changes in all-cause mortality from 2014 to 2016 in 190 Chinese cities**
4. **$PM_{2.5}$-related economic losses from health impacts and changes in total economic losses from 2014 to 2016 in 190 Chinese cities**
5. **References**

1. Source of the health impacts assessment model parameters and the disease data

Table S1 shows the detailed information of those references in the previous research [1], which includes study areas, exposed population, periods, pollutes, and outcomes. In their analysis, they referred to 24 research results, includes reviews [10, 13, 15], summary [16], meta-analysis of the coefficients associating $PM_{2.5}$ exposure and health responses [2-3, 18-20], many of which are the latest research results [2, 4, 16-17, 22, 25], and giving priority to studies performed in China [2-4, 8, 18-20, 23, 25] to maximize regional accuracy.

According to the literature, $PM_{2.5}$ exposure is related to various adverse health impacts. Health impacts of $PM_{2.5}$ were classified to avoid double counting in the successive assessment step. Health impacts were classified into the two categories of mortality and morbidity impacts. Furthermore, they were categorized as either chronic or acute impacts. It should be noted that some health endpoints would occur under both short-term and long-term exposure. For example, the respiratory diseases include both chronic bronchitis and asthma attacks according the 10th version of the International Classification of Diseases (ICD-10) report, so adding the costs of respiratory disease, chronic bronchitis, and asthma attack together will result in double counting.

Table S1. The detailed information of the references in Yin's research

Study area	Population	Period	Pollutants	Outcomes	Field	Adapted from
Beijing-Tianjin-Hebei	All	2009	$PM_{2.5}$	The total health benefit accounted for 1.66%~6.94% of the GDP of this region in 2009.	Meta-analysis of PM-related health impacts, and evaluate health economic loss	[2]
111 cities in China	All	2004	PM_{10}	The total economic cost was estimated at approximately US$ 29,178.7 million.	Meta-analysis on PM-related health impacts, and evaluate health economic loss	[3]
31 cities in China	70,947 middle-aged men and women	1991-2000	TSP; SO_2; NO_x	Each 10 μg/m^3 elevation of TSP was associated with a 0.9% (0.3%, 1.5%) increased risk of cardiovascular mortality	Exposure and mortality	[4]
US	44 senior citizens (age≥ 60)	9:45-14:30	$PM_{2.5}$	An interquartile increase in the 5-day mean $PM_{2.5}$ (6.1 μg/m3) was associated with a 14% increase in CRP (95% CI, –5.4 to 37%) for all individuals and an 81% (95% CI, 21 to 172%) increase for persons with diabetes, obesity, and hypertension.	Associations between ambient PM and markers of systemic inflammation	[5]
204 US urban counties	>200 000	1999-2002	$PM_{2.5}$	Relative risk1.28% (0.78%–1.78%)	Cardiovascular and respiratory hospital admissions associated with short-term exposure to $PM_{2.5}$	[6]
United States metropolitan areas	Ranged 319 000 - 500 000	1982-1998	PM_{10}; $PM_{2.5}$	Cardiovascular causes of death, a 10μ/m^3 increase in fine PM was associated with 8% to 18% increases in mortality risk	Cohort studies: Cardiovascular mortality and long-term exposure to particulate air pollution	[7]

Table S1. *Cont.*

Pearl River Delta	18820000	2006	$PM_{2.5}$; PM_{10}	PM_{10}-related economic loss acounted for 0.09%-0.13% GDP of PRD in 2006	Meta-analysis on PM-related health impacts, and evaluate health economic loss	[8]
Madrid, Spain	——	1995-1998	PM_{10}; SO_2; NO_2; O_3	Relative risk: 1.039 for PM_{10}; 1.029 for SO_2; 1.033 for NO_2; and 1.045 for O_3.	Short-term effects of air pollution on daily asthma	[9]
80 recently published epidemiologic studies	——	Since 1985	Particulate pollution	Observed Particulate pollution related health effects include increased respiratory symptoms, decreased lung function, increased hospitalizations and other health care visits for respiratory and cardiovascular disease, increased respiratory morbidity as measured by absenteeism from work or school or other restrictions in activity, and increased cardiopulmonary disease mortality.	Review: those evaluated effects of particulate pollution at concentrations commonly observed in contemporary cities in the developed world	[10]
Switzerland (Aarau, Basel, Davos, Geneva, Lugano, Montana, Payerne, and Wald)	age 18-60	1991	PM_{10}; TSP; NO_2; O_3	PM_{10}-related RR： 1. 35 (1.11 to 1.65) for chronic phlegm production; 1.27 (1.08 to 1.50) for chronic cough or phlegm production; 1.48 (1.23 to 1.78) for breathlessness during the day; 1.33 (1.14 to 1.55) for breathlessness during the day or at night; and 1.32 (1.18 to 1.46) for dyspnea on exertion.	Long-term ambient air pollution and respiratory symptoms	[11]
23 Apheis cities	age ≥30	1999	$PM_{2.5}$	16,926 premature deaths from all causes, including 11,612 cardiopulmonary deaths and 1901 lung-cancer deaths	Health impact assessment of long-term exposure to $PM_{2.5}$	[12]
Malaysia	——	——	——	See more in [13]	Review of air pollution and health impacts	[13]
29 European cities	——	Short-term	PM_{10}	0.76% (95% confidence interval = 0.47 to 1.05%) in cardiovascular deaths and 0.58% (0.21 to 0.95%) in respiratory deaths	Short-term effects of ambient particles on cardiovascular and respiratory mortality.	[14]

Table S1. *Cont.*

——	——	——	$PM_{2.5}$	Excess risk per 10 μg/m^3 increase in $PM_{2.5}$ exposure was 6% (95% CI 4, 8%) for all-cause and 11% (95% CI 5, 16%) for cardiovascular mortality.	Long-term air pollution exposure and cardio-respiratory mortality: a review	[15]
European	All	Long-term	PM_{10}; $PM_{2.5}$	See more in [16]	Summary: Air pollution public related health burden	[16]
US	age≥20	2004-2005	$PM_{2.5}$	10 μg/m^3 increase in $PM_{2.5}$ exposure (2004: 0.77 [95% CI 0.39–1.25], P 0.001; 2005: 0.81 [0.48–1.07], P 0.001)	The association between $PM_{2.5}$ exposure and diabetes prevalence	[17]
China	All	Long-term	PM_{10}; $PM_{2.5}$	$PM_{2.5}$ concentration increased 10μg/m^3, a 0.40% (95%CI: 0.19%~0.62%) increase for all-course mortality, 1.43% (95%CI: 0.85%~2.01%) increase for respiratory death and 0.53% (95%CI:0.15%~0.90%) increase for cardiovascular death	Meta-analysis: PM-related health impacts	[18]
Shanghai	All	2001	$PM_{2.5}$; PM_{10}; TSP	The total economic cost of health impacts account for 1.03% of GDP of the city.	Meta-analysis: PM-related health impacts, and evaluate health economic loss	[19]
China	All	1990-2002	$PM_{2.5}$; PM_{10}; TSP	See reference [19]	Meta-analysis: Air Particulate Matter -related health impacts	[20]
12 European cities in the APHEA project	——	——	SO_2 and PM_{10}	In western European cities it was found that an increase of 50 μg/m^3 in sulphur dioxide or black smoke was associated with a 3% (2%, 4%) increase in daily mortality and the corresponding figure for PM_{10} was 2% (1% to 3%). In central eastern European cities the increase in mortality associated with a 50 μg/m^3 change in sulphur dioxide was 0.8% (-0.1% to 2.4%) and in black smoke 0.6% (0.1% to 1.1%).	The associations between all-cause mortality and ambient particulate matter and sulphur dioxide	[21]

Table S1. *Cont.*

The Northeastern United States	9.8 million (age ≥ 65)	1999-2010	$PM_{2.5}$	A hazard ratio (HR) of 1.08 (95% CI: 1.05, 1.11) for dementia, an HR of 1.15 (95% CI: 1.11, 1.19) for AD, and an HR of 1.08 (95% CI: 1.04, 1.12) for PD admissions per 1-µg/m3 increase in annual $PM_{2.5}$ concentrations.	Long-term $PM_{2.5}$ Exposure and Neurological Hospital Admissions	[22]
Hongkong	69716 admissions	200-2005	NO_X; SO_2; O_3; $PM_{2.5}$; PM_{10}	The relative risks (RR) for hospitalization for every 10 µg/m^3 increase in NO_2, O_3, PM_{10} and $PM_{2.5}$ were 1.028, 1.034, 1.019 and 1.021, respectively	Ambient air pollutants and hospitalization rates for asthma	[23]
50 states in US	500000 adults	1982-1998	Particulate pollution	Each 10 µg/m^3 elevation in fine particulate air pollution was associated with approximately a 4%, 6%, and 8% increased risk of all-cause, cardiopulmonary, and lung cancer mortality	Long-term exposure to fine particulate air pollution and all-cause, lung cancer, and cardiopulmonary mortality	[24]
China	All	2012	Air pollution	See Table S3	Baseline incidence rates	[25]

As a result, Yin utilized the ICD-10 report to avoid double counting of $PM_{2.5}$ health impacts. According to the ICD-10 disease classification, there are six main categories of diseases related to the $PM_{2.5}$ pollutants: circulatory system disease; respiratory disease; endocrine, nutritional and metabolic diseases; neoplasms; mental and behavioral disorder and nervous system diseases. The main ICD-10 health endpoints related to $PM_{2.5}$ are reported in Table S2 together with their ICD-10 codes and the sources reporting their epidemiological evidence.

Table S2. $PM_{2.5}$-related health endpoints according to the ICD-10 reports and literature evidence [1]

**Mortality**				
	**Diseases of the circulatory system [IX]**	**Neoplasms [II]**	**Diseases of the respiratory system [X]**	
Chronic mortality	Cardiovascular Mortality [I51.6] [7]<br>Cardiopulmonary Mortality [I27.9] [12]	Lung cancer mortality [C34.9] [24]	Respiratory mortality [J00-J99] [15]	
Acute mortality	Cardiovascular mortality [I51.6] [14,21]		Respiratory Mortality [J00-J99] [14]	
**Morbidity**				
	**Diseases of the circulatory system [IX]**	**Diseases of the respiratory system [X]**		
Acute morbidity	Cardiovascular disease [I51.6] [6]	Respiratory diseases [6]<br>Acute bronchitis [J20-J22] [8]<br>Asthma attacks [J45] [9]		
	**Diseases of the respiratory system [X]**	**Endocrine, nutritional and metabolic diseases [IV]**	**Diseases of the nervous system [VI]**	**Mental and behavioral disorders [V]**
Chronic morbidity	Chronic bronchitis [J41] [11]	Diabetes [E11] [5, 17]	Alzheimer [G30.1] [22]	Dementia [F03] [22]
	Asthma attacks [J45] [9]	Obesity [E66] [5]	Parkinson [22]	Mild cognitive disorder[F06.7] [16]<br>Childhood autism [F84.0] [16]
Undefined	Restricted activity day [10, 13]	Internal medicine outpatient visits [10]	Pediatric outpatient visits [10]	

Notes: An empty cell means that no health endpoint has been reported in that category according to the authors' knowledge; "[code]" denotes the disease classification code according to the ICD-10 report.

Moreover, based on the health impact identification and classification, $PM_{2.5}$ health impacts were estimated for cardiovascular mortality, cardiopulmonary mortality, lung cancer mortality, respiratory mortality, chronic bronchitis, cardiovascular hospitalization, acute bronchitis, and asthma attacks. Exposed coefficients in Yin's study were selected according to those studies (Table 1), different health endpoints selected the latest research results in their field separately, as shown in Table S3. If there were several studies describing the exposure-response function for the same health endpoint, they used the pooled estimate to get the mean and 95 percent confidence interval (CI) of the coefficient. This meta-analysis method was based on the variance weighted average across the results of studies with available quantitative effect estimates (coefficients or relative risks). Studies with lower standard errors had more weight in the resulting joint estimate. In addition, some studies using TSP and $PM_{2.5}$ for exposure assessment were also included in the analysis. The following conversion factors were applied for different particulate matter indicators: PM_{10}=TSP×0.65 and $PM_{2.5}$=PM_{10}×0.65 [20]

Table S3. $PM_{2.5}$ exposure-response coefficients and baseline incidence rates for the analyzed health endpoints [1]

Health impact	Exposure time	Population	β_i (CI=95%)	E_{0i}
All-cause mortality	Long-term	All	9.00E-04 (0, 1.8E-03) [4]	6.14E-03 [25]
Cardiovascular mortality	Long-term	All	5.30E-04 (1.50E-04, 9.00E-04) [18]	2.69E-03 [25]
Respiratory mortality	Long-term	All	1.43E-03 (8.50E-04, 2.01E-03) [18]	5.80E-04 [25]
Lung cancer mortality	Long-term	Age≥15	3.40E-03 (0.00E+00, 7.10E-03) [4]	4.97E-04 [25]
Chronic bronchitis	Long-term	All	2.7E-03 (7.62E-04, 4.64E-03) [3,19,20]	6.94E-03 [2]
Cardiovascular hospital admission	Short-term	All	6.80E-04 (4.30E-04, 9.30E-04) [2]	5.46E-03 [2]
Asthma attack	Short-term	All	2.10E-03 (1.45E-03, 2.74E-03) [23]	9.40E-03 [2]
Acute bronchitis	Short-term	All	7.90E-03 (2.70E-03, 1.30E-02) [8]	3.80E-02 [2]

Lastly, considering that both of our study areas belong to China, and their article has just been published, our research was conducted at a similar time in terms of timeliness. Taking the above factors into account, we believe that their results are reliable, and cited their meta-analysis results as the source of disease data in this study.

2. Assumption cells and forecast cells used for uncertainty analysis

The uncertainty analysis covered $PM_{2.5}$ exposure concentration, exposure–response coefficients, and health-related economic loss per case. In addition, AHC values were calculated based on the per capita GDP, and because the uncertainty of this estimate was unknown, the

uncertainty of economic losses from mortality estimated using the AHC approach was not included in this study.

First, we built assumption cells (see Table 4) in Crystal ball software according to the above uncertainty analysis, and their probability distribution obtains from the results of previous studies (see Table S4).

Table S4. Assumption cells and assigned probability distribution [1]

Assumption cells	Health impact	Probability distribution	Values Mean (Std)	Units
Exposure-response coefficients β		Lognormal	See S1	m³/μg
Exposure concentration C		Lognormal		μg/m³
Economic loss for premature death j	All-cause mortality	Lognormal	VSL: 232,000 (1.43)<br>AHC: 132,000 (1)	US $ in 2012
Cost j (diseases)	Chronic bronchitis	Lognormal	VSL: 13,000 (1.43)<br>AHC: 7,000 (1)	US $ in 2012
Cost j (diseases)	Cardiovascular hospital admission	Lognormal	COL:1600(1.23)	US $ in 2012
Cost j (diseases)	Asthma attack	Lognormal	WTP:7(1.27)	US $ in 2012
Cost j (diseases)	Acute bronchitis	Lognormal	WTP:9(1.25)	US $ in 2012

Then, we built forecasting cells (see Table S5) with equations (to calculate health economic loss by VSL) and dependent assumption cells.

Table S5. Forecasting cells with equations and dependent assumption cells

Forecasting cells	Equation	Assumption cells
Total economic loss	$EC_{ij} = Pop_i * E_{0i} * e^{\beta(C_i - C_0)} * Cost_j$	Exposure-response coefficients β<br>Exposure concentration C<br>$Cost_j$ Economic loss for health endpoint j (diseases)

3. $PM_{2.5}$-related health impacts and changes in all-cause mortality from 2014 to 2016 in 190 Chinese cities

See Appendix S3：$PM_{2.5}$-related health impacts and changes in all-cause mortality from 2014 to 2016 in 190 Chinese cities.xls

S4. $PM_{2.5}$-related economic losses from health impacts and changes in total economic losses from 2014 to 2016 in 190 Chinese cities

See Appendix S4：$PM_{2.5}$-related economic losses from health impacts and changes in total economic losses from 2014 to 2016 in 190 Chinese cities.xls

5. References

[1] Yin, H.; Pizzol, M.; Xu, L. External costs of PM2.5 pollution in Beijing, China: Uncertainty analysis of multiple health impacts and costs. *Environ. Pollut.* **2017**, 356-369. DOI: 10.1016/j.envpol.2017.02.029

[2] Huang, D.; Zhang, S. Health benefit evaluation for PM2.5 pollution control in Beijing-Tianjin-Hebei region of China. *China. Environ. Sci.* **2013**, *1*, 166-174. (in Chinese) Available online: http://zghjbh.com.cn/uploadfile/2016/0615/20160615101327987.pdf

[3] Zhang, M.; Song, Y.; Cai, X.; Zhou, J. Economic assessment of the health effects related to particulate matter pollution in 111 Chinese cities by using economic burden of disease analysis. *Environ. Manage.* **2008**, *4*, 947-954. DOI: 10.1016/j.jenvman.2007.04.019

[4] Cao, J.; Yang, C.; Li, J.; Chen, R.; Chen, B.; Gu, D.; Kan, H. Association between long-term exposure to outdoor air pollution and mortality in China: a cohort study. *J. Hazard. Mater.* **2011**. *186,* 1594-1600. DOI: 10.1016/j.jhazmat.2010.12.036

[5] Dubowsky, S.D.; Suh, H.; Schwartz, J.; Coull, B. A.; Gold, D. R. Diabetes, obesity, and hypertension may enhance associations between air pollution and markers of systemic inflammation. *Environ. Health Perspect.* **2006**. 992-998. http://www.jstor.org/stable/3651767

[6] Dominici, F.; Peng, R. D.; Bell, M. L.; Pham, L.; McDermott, A.; Zeger, S. L.; Samet, J. M. Fine particulate air pollution and hospital admission for cardiovascular and respiratory diseases. *Jama* **2006**. *295*, 1127-1134. DOI: 10.1001/jama.295.10.1127

[7] Pope, C. A.; Burnett, R. T.; Thurston, G. D.; Thun, M. J.; Calle, E. E.; Krewski, D.; Godleski, J. J. Cardiovascular mortality and long-term exposure to particulate air pollution epidemiological evidence of general pathophysiological pathways of disease. *Circ.* **2004**. *109*, 71-77. DOI: 10.1161/01.CIR.0000108927.80044.7F

[8] Liu, X.; Xie, P.; Liu, Z.; Li, T.; Zhong, L.; Xiang, Y. Economic assessment of acute health impact due to inhalable particulate air pollution in the Pearl River Delta. *J. Peking Uni.* (Natural Science, in Chinese). **2010**, *46*, 829-834

[9] Galan, I.; Tobias, A.; Banegas, J.; Aranguez, E. Short-term effects of air pollution on daily asthma emergency room admissions. *Eur. Respir. J.* **2003,** *22,* 802-808. DOI: 10.1183/09031936.03.00013003

[10] Pope III, C.; Bates, D. V.; Raizenne, M. E. Health effects of particulate air pollution: time for reassessment? *Environ. Health Perspect.* **1995**. *103*, 472-480

[11] Zemp, E.; Elsasser, S.; Schindler, C.; Kunzli, N. et al. Long-term ambient air pollution and

respiratory symptoms in adults (SAPALDIA study). *Am. J. Respir. Crit. Care Med.* **1999.** *159,* 1257-1266. DOI:10.1164/ajrccm.159.4.9807052

[12] Boldo, E.; Medina, S.; Le Tertre, A.; Hurley, F.; Mücke, H. G.; Ballester, F.; Aguilera, I. Apheis: Health impact assessment of long-term exposure to PM2.5 in 23 European cities. *Eur. J. Epidemiol.* **2006**. *21*, 449-458. DOI: 10.1007/s10654-006-9014-0

[13] Afroz, R.; Hassan, M. N.; Ibrahim, N. A. Review of air pollution and health impacts in Malaysia. *Environ. Res.* **2003**. *92*, 71-77. DOI:10.1016/S0013-9351(02)00059-2

[14] Analitis, A.; Katsouyanni, K.; Dimakopoulou, K.; Samoli, E.; Nikoloulopoulos, A. K.; Petasakis, Y.; Touloumi, G.; Schwartz, J.; Anderson, H. R.; Cambra, K. Short-term effects of ambient particles on cardiovascular and respiratory mortality. *Epidemiol.* **2006**. *17*, 230-233. DOI: 10.1097/01.ede.0000199439.57655.6b

[15] Hoek, G.; Krishnan, R. M.; Beelen, R.; Peters, A.; Ostro, B.; Brunekreef, B.; Kaufman, J. D. Long-term air pollution exposure and cardio-respiratory mortality: a review. *Environ. Health.* **2013**. *12*, 43-57. http://www.ehjournal.net/content/12/1/43

[16] Holgate, S.; Grigg, J.; Agius, R.; Ashton, J.R.; Cullinan, P.; Exley, K.; Fishwick, D.; Fuller, G.; Gokani, N.; Griffiths, C. Every breath we take: The lifelong impact of air pollution, Report of a working party. R. Coll. Physicians. **2016**. London. *ISBN: 978-1-86016-567-2.* Available on: https://www.rcplondon.ac.uk/projects/outputs/every-breath-we-take-lifelong-impact-air-pollution

[17] Pearson, J. F.; Bachireddy, C.; Shyamprasad, S.; Goldfine, A. B.; Brownstein, J. S. Association between fine particulate matter and diabetes prevalence in the US. *Diabetes care.* **2010**. *33,* 2196-2201. DOI: 10.2337/dc10-0698

[18] Peng, X.; Xiaoyun, L.; Zhaorong, L.; Tiantian, L.; Yuhua, B. Study of exposure-response relationship of air particulate pollution in China. *China Environ. Sci.* (in Chinese) **2009**. *29,* 1034-1040.

[19] Kan, H.; Chen, B. Particulate air pollution in urban areas of Shanghai, China: health-based economic assessment. *Sci. Total Environ.* **2004**. *322,* 71-79. DOI: 10.1016/j.scitotenv.2003.09.010

[20] Kan, H.; Chen, B.; Chen, C.; Wang, B.; Fu, Q. Establishment of exposure-response functions of air particulate matter and adverse health outcomes in China and worldwide. *Biomed. Environ. Sci.* **2005**. *18*, 159.

[21] Katsouyanni, K.; Touloumi, G.; Spix, C.; Schwartz, J.; Balducci, F.; Medina, S.; Rossi, G.; Wojtyniak, B.; Sunyer, J.; Bacharova, L. Short term effects of ambient sulphur dioxide and particulate matter on mortality in 12 European cities: results from time series data from the APHEA project. *Bmj* **1997.** *314*, 1658. DOI: 10.1136/bmj.314.7095.1658

[22] Kioumourtzoglou, M. A.; Schwartz, J. D.; Weisskopf, M. G.; Melly, S. J.; Wang, Y.; Dominici, F.; Zanobetti, A. Long-term PM2.5 exposure and neurological hospital admissions in the Northeastern United States. *Environ. Health Perspect.* **2016.** *124,* 23. DOI: 10.1289/ehp.1408973

[23] Ko, F.; Tam, W.; Wong, T.; Lai, C.; Wong, G.; Leung, T.; Ng, S.; Hui, D. Effects of air pollution on asthma hospitalization rates in different age groups in Hong Kong. *Clin. Exp. Allergy.* **2007**. *37*, 543-551. DOI: 10.1007/s11869-013-0195-x

[24] Pope III, C. A.; Burnett, R. T.; Thun, M. J.; Calle, E. E.; Krewski, D.; Ito, K.; Thurston, G. D.

Lung cancer, cardiopulmonary mortality, and long-term exposure to fine particulate air pollution. *Jama* **2002**. *287,* 1132-1141.DOI: 10.1001/jama.287.9.1132
[25] NHFPC. China Health Statistical Yearbook. Pecking Union Medical College Press, **2013**. Beijing (National Health and Family Planning Commission).
